# Environmental Enrichment Promotes Plasticity and Visual Acuity Recovery in Adult Monocular Amblyopic Rats

**DOI:** 10.1371/journal.pone.0034815

**Published:** 2012-04-11

**Authors:** Paola Tognini, Ilaria Manno, Joyce Bonaccorsi, Maria Cristina Cenni, Alessandro Sale, Lamberto Maffei

**Affiliations:** 1 Laboratory of Neurobiology, Scuola Normale Superiore, Pisa, Italy; 2 Institute of Neuroscience CNR, Pisa, Italy; Dalhousie University, Canada

## Abstract

Loss of visual acuity caused by abnormal visual experience during development (amblyopia) is an untreatable pathology in adults. In some occasions, amblyopic patients loose vision in their better eye owing to accidents or illnesses. While this condition is relevant both for its clinical importance and because it represents a case in which binocular interactions in the visual cortex are suppressed, it has scarcely been studied in animal models. We investigated whether exposure to environmental enrichment (EE) is effective in triggering recovery of vision in adult amblyopic rats rendered monocular by optic nerve dissection in their normal eye. By employing both electrophysiological and behavioral assessments, we found a full recovery of visual acuity in enriched rats compared to controls reared in standard conditions. Moreover, we report that EE modulates the expression of GAD67 and BDNF. The non invasive nature of EE renders this paradigm promising for amblyopia therapy in adult monocular people.

## Introduction

Amblyopia (also called lazy eye) is a pathological reduction in visual perceptual abilities due to a defective sensory experience during development. This condition, which has a prevalence of about 3% of the total world population [1], derives from a functional imbalance between the two eyes, typically caused by unilateral congenital cataract, anisometropia or strabismus, resulting in an ocular dominance shift toward the normal eye in the primary visual cortex and a prominent loss of visual acuity (VA) in the affected eye [2], [3]. In animal models, amblyopia can be artificially induced by imposing a long-term reduction of inputs from one eye by lid suture (monocular deprivation, MD) [4], [5], [6], [7]. While VA and ocular dominance can recover to normal if a normal visual experience is restored early in development during the so-called critical period, no recovery is instead observed in the adult, due to a dramatic reduction of plasticity levels which occurs in the visual cortex at the end of the critical period [8], [9].

One case of particular interest is that of amblyopic patients who loose their better eye, thus becoming severely visually impaired. While it has been reported that adult amblyopes can occasionally improve VA following loss of vision in the fellow eye either due to a sudden accident or to progressive development of ocular illnesses [10], [11], [12], [13], [14], which factors promote improvement under these circumstances are still totally unknown. Besides its clinical importance, this condition is also highly relevant from a basic research perspective, representing a useful model to investigate visual cortex plasticity when binocular competition is completely suppressed. Starting from the pioneering work by Hubel and Wiesel and, subsequently, as demonstrated by Stryker and colleagues, it has been shown that visual cortical plasticity operates through a competitive interaction between inputs from the two eyes [15], [16], [17], [18]. In contrast, much less evidence has been reported in favor of experience-dependent plasticity processes in the total absence of binocular interactions between the two eyes. Experiments conducted over thirty years ago on monocularly deprived cats showed that the effects of MD on cat striate cortex cells can be partially reversed after the critical period by enucleation of the experienced eye [19]. However, whether VA recovery is possible under these conditions was never investigated.

We recently reported that environmental enrichment (EE), a milieu characterized by increased social interactions, sensory-motor activity and exploratory behavior, is highly effective in enhancing plasticity in the primary visual cortex of adult amblyopic rats, promoting a full recovery of VA and ocular dominance, accompanied by a marked reduction of intracortical GABAergic inhibition [7]. Since EE is a totally non-invasive procedure with a high potential to be applied to human patients, here we asked whether exposure to EE could also be employed to trigger recovery of vision in adult amblyopic rats rendered monocular by means of optic nerve dissection (OND) in their normal eye.

## Materials and Methods

### Animal treatment and surgical procedures

All experiments were performed on Long-Hevans hooded rats in accordance with the Italian Ministry of Public Health guidelines for care and use of laboratory animals. Specifically, the experiments described in this study were authorized by the Italian Ministry of Health via decree # 185/2009-B, released on November 4, 2009. All experiments respected the welfare of animals and have been conducted strictly according to the legal and ethical requirements demanded by law. The Institute of Neuroscience CNR has been authorized by the Italian Ministry of Public Health to the use of animals for scientific purposes (authorization # 129/2000-A, December 13, 2000). The lowest degree of neuropsychological sensitivity, pain, suffering and distress have been ensured by the employment of approved methods of anaesthesia and animal handling and by the application of specific anti-pain drugs whenever required. The experimental activity was guided by the principle of the Three R's (Replacement, Reduction and Refinement).

Rats lived in an animal house with a temperature of 21°C, 12/12 light/dark cycle and food and water available ad libitum. To assess the effects of long term monocular deprivation (MD), postnatal day (P) 21 rats were anesthetized with avertin (1 ml/hg) and monocularly deprived through eyelid suturing until adult age (P60). Rats showing occasional lid reopening (observed with a surgical microscope) were not included in the experiments. The long-term deprived eye of adult amblyopic rats was re-opened using thin scissors, under anaesthesia, while the optic nerve of the other eye was dissected mechanically with thin surgical forceps. The crush was performed intracranially, 3 mm from the posterior pole of the eye; special attention was taken to avoid mechanical damage of the retinal artery. Great care was taken during the first days after surgery to prevent inflammation or infection in the previously deprived eye through topical application of antibiotic and cortisone.

After OND, the rats were divided into two groups. A group was reared in standard laboratory cages (30×40×20 cm, standard condition SC, OND-SC) housing each 2 rats, and a second group of animals was reared in environmental enrichment (EE, OND-EE). For comparison, a further group of untreated rats reared in SC (naïve animals) was also included in the analysis. Enriched environment consisted of large wire netting cages (60×50×80 cm) with three floors containing several food hoppers, running wheels to allow physical activity, and differently shaped objects (tunnels, shelters, stairs) that were completely substituted with others once a week. Every cage housed 6–8 rats.

After three weeks of EE or SC, the VA of the re-opened eye was analyzed by *in vivo* electrophysiology.

### In vivo electrophysiology

The animals (OND-SC, n = 6; OND-EE, n = 6; naïve, n = 8) were anesthetized with urethane (0.7 ml/hg; 20% solution in saline) by i.p. injection and placed in a stereotaxic apparatus. Body temperature was continuously monitored and maintained at ∼37°C by a thermostated electric blanket during the experiment. An ECG was continuously monitored. A hole was drilled in the skull, corresponding to the binocular portion of the primary visual cortex (binocular area Oc1B) contralateral to the previously deprived eye. After exposure of the brain surface, the dura was removed, and a micropipette (2 MΩ) filled with NaCl (3 M) was inserted into the cortex 5 mm from λ. The re-opened eye was fixed and kept open by means of adjustable metal rings surrounding the external portion of the eye bulb. We measured VA using visual evoked potentials (VEPs). To record VEPs, the electrode was advanced at a depth of 100 or 400 µm within the cortex. At these depths, VEPs had their maximal amplitude. Signals were band-pass-filtered (0.1–100 Hz), amplified, and fed to a computer for analysis, as described previously [20]. Briefly, at least 128 events were averaged in synchrony with the stimulus contrast reversal. Transient VEPs in response to abrupt contrast reversal (0.5 Hz) were evaluated in the time domain by measuring the peak-to-baseline amplitude and peak latency of the major positive or negative component. Visual stimuli were horizontal sinusoidal gratings of different spatial frequencies and contrast, generated by a VSG2/2 card running custom software and presented on a monitor (20×22 cm; luminance 15 cd/m^2^) positioned 20 cm from the rat's eyes and centred on the previously determined receptive fields. Visual acuity was obtained by extrapolation to zero amplitude of the linear regression through the last four to five data points in a curve where VEP amplitude is plotted against log spatial frequency.

### Behavioral assessment of visual acuity

We measured VA of the fellow (not deprived) eye in long-term monocularly deprived rats at P50–P60, before performing OND. Next, we measured VA of the formerly deprived eye (long-term deprived), after OND and three weeks of SC (n = 3) or EE (n = 4) rearing. Therefore, VA measurement of the formerly deprived eye was completed when the animals were about P90–100. To measure VA, we used the visual water box task [21], which trains animals to first distinguish a low (0.1 cycles per degree; c/deg) spatial frequency vertical grating from grey, and then tests the limit of this ability at higher spatial frequencies. The apparatus consists of a trapezoidal-shaped pool with two panels placed side by side at one end. A midline divider is extended from the wide end of the pool into the middle, creating a maze with a stem and two arms. The length of the divider sets the choice point and effective spatial frequency. An escape platform is placed below the grating. Animals are released from the centre at the end of the pool opposite the panels. The position of the grating and the platform is alternated in a pseudorandom sequence over training trials while the rats are shaped to swim towards the grating in one of the maze arms. A trial is recorded as incorrect if an animal enters the arm without the platform. Animals are removed from the pool when they find the platform. Once 80% accuracy is achieved, the limit of the discrimination is estimated by increasing the spatial frequency of the grating. Visual acuity has been taken as the spatial frequency corresponding to 70% of correct choices on the sigmoidal function fitting the psychometric function. During each session, the experimenter was blind to the experimental group.

### Immunohistochemistry

Animals (BDNF staining: OND-SC, n = 6, OND-EE, n = 7; GAD67 staining: OND-SC, n = 4, OND-EE, n = 8) were deeply anesthetized with chloral hydrate and perfused transcardially with PBS followed by fixative (4% paraformaldehyde, 0.1 M sodium phosphate, pH 7.4, PB). Brains were gently removed, post-fixed for 4–6 hours before being cryoprotected by immersion in 30% sucrose. Coronal sections (50 µm) from the cortex were cut on a microtome and collected in PBS.

BDNF staining. Free-floating sections were incubated for 1 hour in a blocking solution (10% BSA, 0.3% Triton X-100, in PBS, pH 7.4). Sections were incubated overnight at 4°C in a solution of chicken polyclonal anti-BDNF antibody (1∶400, Promega). Primary antibody was revealed with biotinylated donkey anti-chicken (1∶200, Promega) followed by fluorescein-conjugated extravidin (1∶300, Sigma). Sections of the two experimental groups were reacted together within the same immunohistochemistry session. Images were acquired at 20× magnification (NA = 0.7; field 707×707 µm acquired at 1024×1024 pixels) using a confocal Leica microscope and imported to the image analysis software MetaMorph to analyse the number of BDNF positive cells. To compare different specimens, the parameters of acquisition (laser intensity, gain, offset) were optimized at the start and then held constant throughout image acquisition. Counts were done on the entire thickness of Oc1B and normalized to the Oc1B area (mm^2^). For each animal, at least five Oc1B sections were analyzed.

GAD67 staining. Free-floating sections were incubated for 1–2 hour in a blocking solution (10% BSA, 0.3% Triton X-100, in PBS, pH 7.4). Sections were then incubated overnight at 4°C in a solution of mouse monoclonal anti-GAD67 antibody (1∶1000, Chemicon MAB5406), 1% BSA, 0,3% Triton X-100 in PBS 1×. Primary antibody was revealed with Alexa 568 antimouse (1∶400, Molecular Probes, Invitrogen). Images were acquired and analyzed with the same method described for BDNF staining.

## Results

**Figure 1:**
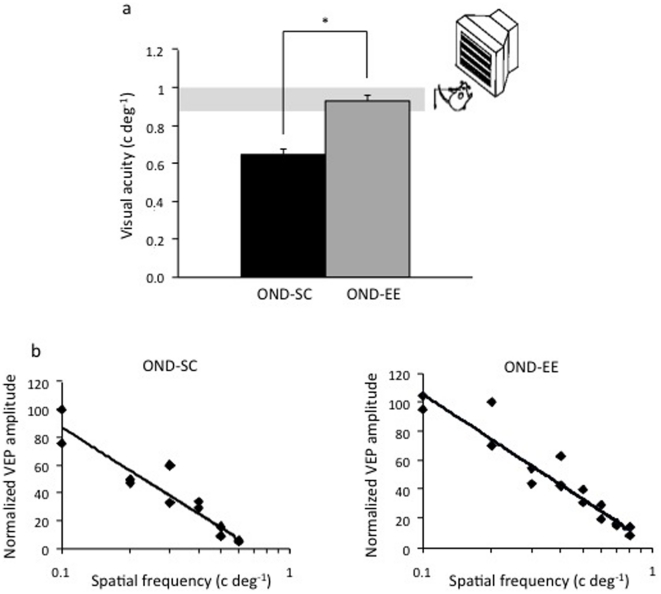
Environmental enrichment promotes recovery of visual acuity in adult amblyopic monocular rats. a) Electrophysiological assessment of visual acuity (VA) showed a significant difference between OND-EE rats and OND-SC animals and between OND-SC rats and untreated controls (grey shadow in the graph), but not between untreated controls and OND-EE rats (One Way ANOVA p<0.001, post-hoc Holm-Sidak test OND-SC vs. OND-EE and untreated controls p<0.05, other comparisons were not significantly different). Insert shows a schematic representation of the apparatus used for VEP recording. b) Representative examples of VA estimates for the previously deprived eye in OND-SC and OND-EE animals. Percentage of normalized VEP amplitude is plotted against log spatial frequency. Visual acuity is obtained by extrapolation to zero amplitude of the linear regression through the data points in a curve where VEP amplitude is plotted against log spatial frequency. *, statistical significance; error bars represent s.e.m.

Rats were deprived at P21 and raised under standard conditions until adulthood. At this time, the deprived eye was reopened and rats rendered monocular by OND of their fellow eye. Then, the animals were transferred to either standard conditions (OND-SC) or to EE (OND-EE), for three weeks. At the end of the differential rearing period, VA of the amblyopic eye was assessed using *in vivo* electrophysiological recordings of VEPs from the visual cortex contralateral to the long-term deprived eye. We found that the VA of OND-EE rats (0.93±0.03 c/deg) was significantly higher than that of OND-SC rats (0.65±0.03 c/deg) and was not different from that of untreated naïve animals (0.90±0.02 c/deg) (Fig. 1a, b).

**Figure 2:**
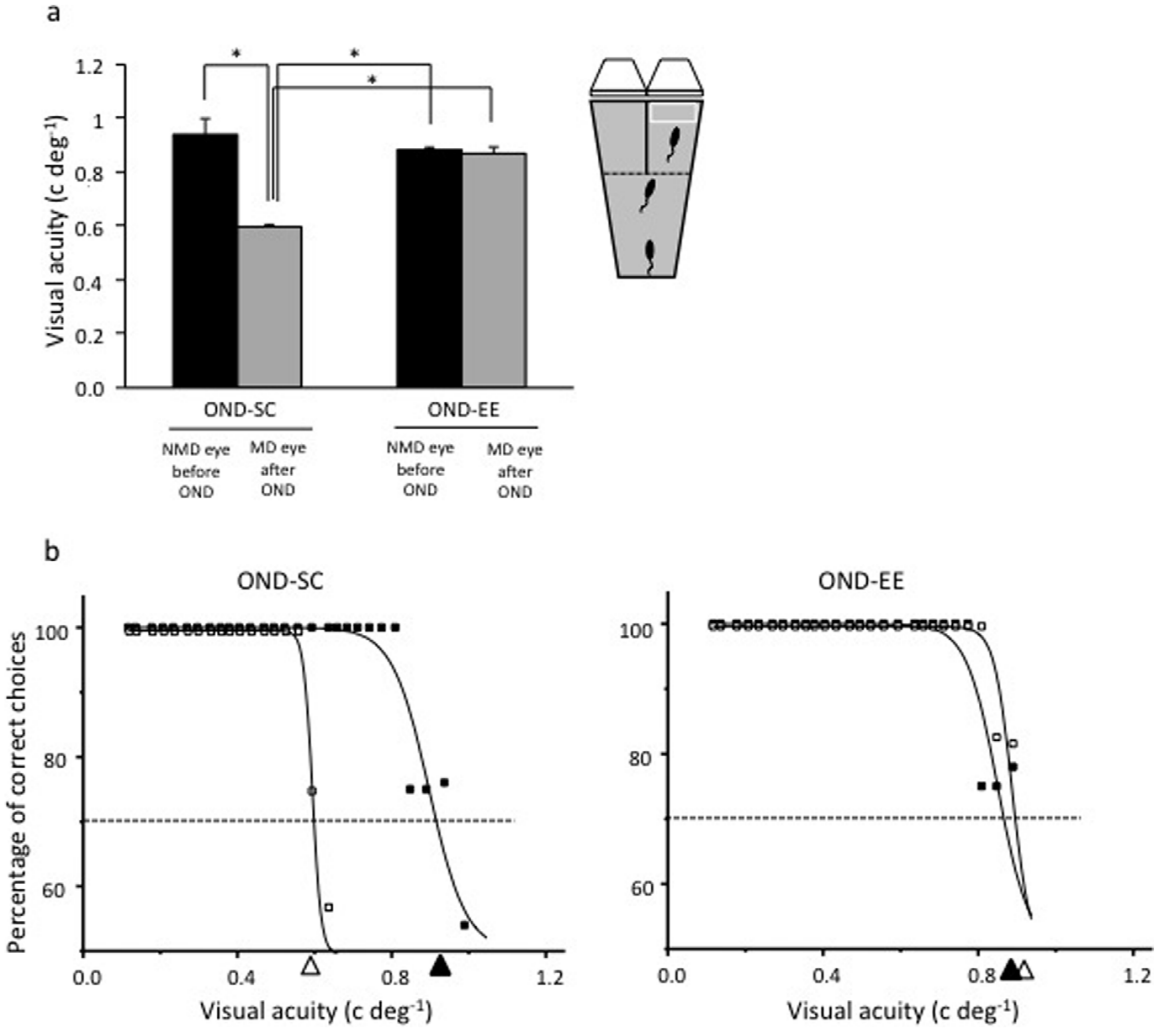
Environmental enrichment promotes recovery of behavioural perceptual abilities in adult amblyopic monocular animals. a) Statistical analysis showed that the visual acuity (VA) of the previously deprived eye was not different from that of the other eye (fellow eye) in OND-EE rats (paired t-test, p = 0.51); a statistical difference was instead present between the VA of the previously deprived eye and that of the fellow eye in OND-SC animals (paired t-test, p<0.05). Insert shows a schematic representation of the visual water-box task, the apparatus used for behavioral assessment of visual acuity. b) Representative examples of behavioral VA estimates for both the previously deprived and the fellow eye in OND-SC (left) and OND-EE (right) animals. Visual acuity is obtained by extrapolation to 70% of correct choices on the sigmoidal function fitting the psychometric function in which the percentage of correct choices is plotted against zero spatial frequency. *, statistical significance; error bars represent s.e.m.

In amblyopia, recovery of a proper cellular response to visual stimuli in the primary visual cortex is useless if such effect does not transfer to behavioral perceptual abilities. Therefore, we repeated VA assessments also by using a standard behavioral task, the visual water-box task [21], [22]. The behavioral measure of VA completely confirmed the electrophysiological data: a marked recovery was evident in OND-EE animals (0.87±0.05 c/deg) with respect to OND-SC rats (0.60±0.01 c/deg) (Fig. 2a, b).

**Figure 3:**
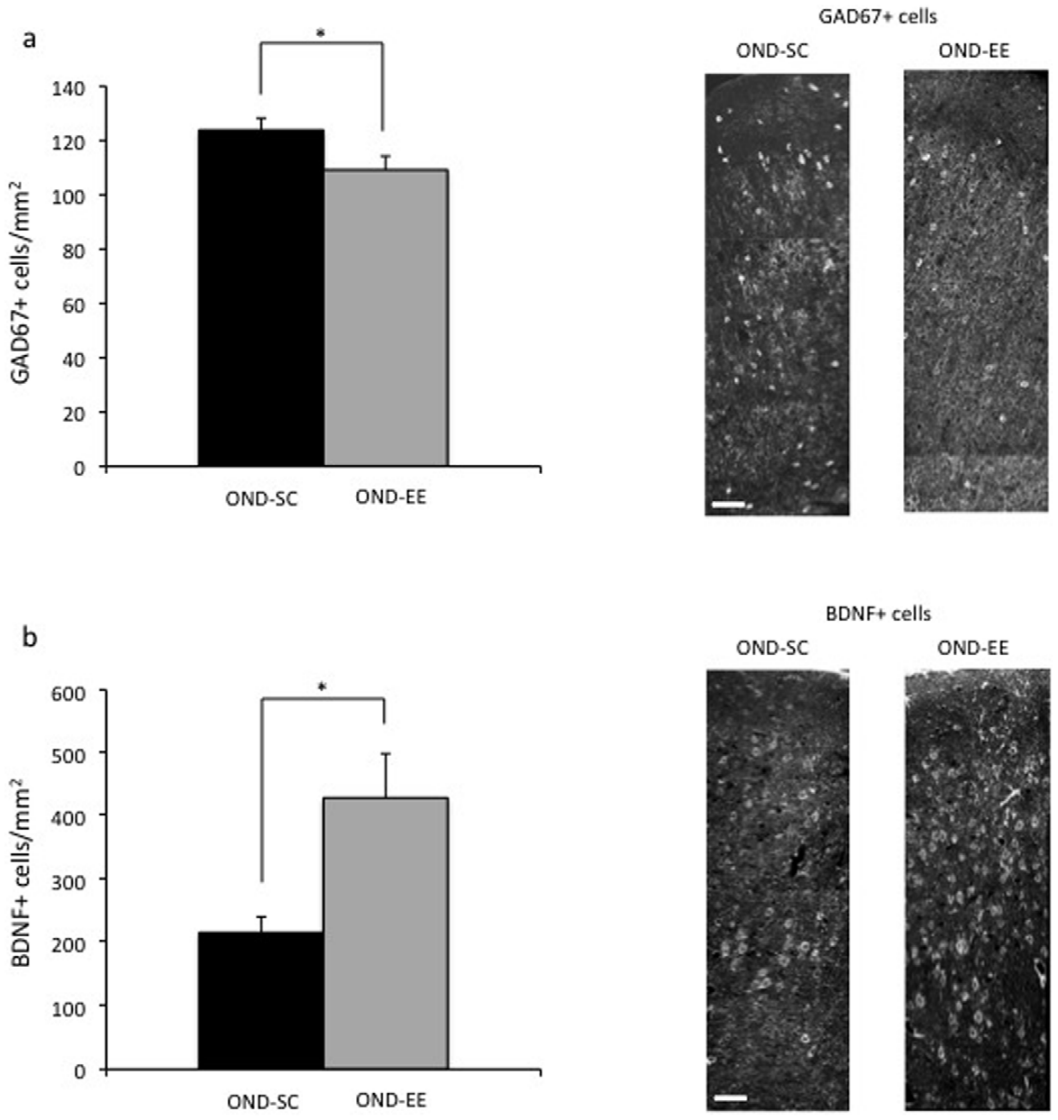
Environmental enrichment induces changes of plasticity factors in the visual cortex of adult amblyopic monocular rats. a) EE decreases GAD67 expression in the visual cortex contralateral to the previously deprived eye. The number of GAD67+ cells was statistically lower in the visual cortex of OND-EE with respect to OND-SC rats (t-test, p<0.001). b) EE increases BDNF expression in the visual cortex contralateral to the previously deprived eye. The number of BDNF+ cells was statistically higher in the visual cortex of OND-EE with respect to OND-SC rats (Mann-Whitney Rank sum test, p = 0.035). *, statistical significance; calibration bar = 40 µm; error bars represent s.e.m.

Previous studies demonstrated that the maturation of inhibitory circuits [8] is one of the most important factor responsible for the closure of critical period plasticity in the visual system and that EE can restore plasticity in the adult brain through a reduction of intracortical inhibition [7]. To assess whether the restored plasticity detected after three weeks of EE in OND amblyopic rats was accompanied by a change in the GABAergic system, glutamic acid decarboxylase 67 (GAD67) protein levels were measured in the visual cortex of OND-EE and OND-SC animals by using immunohistochemistry. The number of GAD67-positive cells normalized to the area of Oc1B (GAD67+) was significantly decreased in OND-EE animals compared to OND-SC rats (OND-SC GAD67+ = 123.81±4.41 cells/mm^2^; OND-EE GAD67+ = 109.16±5.09 cells/mm^2^; Fig. 3a).

Finally, we investigated whether exposure to EE also resulted in an increase of the neurotrophin BDNF, which is known to be involved in the development and plasticity of the visual system [23] and which has also been linked to visual acuity recovery after deprivation [7], [24], [25]. Immunoistochemistry analysis revealed a significant increase in BDNF positive cells normalized to the area of Oc1B (BDNF+) in the visual cortex of OND-EE animals compared to OND-SC rats (OND-SC BDNF+ = 216.2±24.99 cells/mm^2^; OND-EE BDNF+ = 429.22±70.09 cells/mm^2^; Fig. 3b).

## Discussion

In the present work we addressed the possibility to rescue VA in amblyopic adult rats exposed to EE immediately after silencing of retino-thalamic projections of the fellow (non amblyopic) eye due to OND. We observed that, while no spontaneous rescue of the VA impairment was detected in rats reared under standard environmental conditions, a full recovery of VA in the amblyopic eye was achieved in monocular adult rats after three weeks of EE. Moreover, we also showed that this effect is accompanied by modulation of two classic factors critically involved in visual cortex plasticity, BDNF and the GABAergic system. The lower number of GAD67+ cells after exposure to EE indicates an overall reduction of GABAergic inhibition in the visual cortex of OND-EE rats, in agreement with our previous results showing a reduced GABA release in the visual cortex of enriched amblyopic animals [7]. It is instead very unlikely that this effect is due to a change in the total number of inhibitory neurons, as this would imply increased cell death rates induced by EE.

The possibility to induce plasticity in the visual cortex of adult amblyopic monocular rats is intriguing since after OND of the fellow eye binocular interactions in the visual cortex are suppressed. According to a competition-based model, the stronger inputs from the fellow eye could mask activity in the weaker connections from the amblyopic eye. Thus, the connections from the long-term deprived eye may be suppressed rather than completely eliminated, making it possible to unmask them after loss of the fellow eye.

Plasticity in monocular amblyopic rats is also consistent with the predictions of BCM theory, proposed by Bienenstock, Cooper and Munro [26]. In contrast to competition-based models, the BCM model introduces a homosynaptic-learning rule. Therefore, a change in synaptic strength in one input upon a cell may occur without alteration in another set. The model also incorporates a sliding “modification threshold” for synaptic modification that varies depending on the average postsynaptic activity over time. Synaptic strengthening occurs when presynaptic activity causes postsynaptic activity to exceed the modification threshold. According to the BCM theory, inputs from the two eyes interact through changes in the average activity of the postsynaptic cell [27]. In our model, OND of the fellow eye can lead to a strong reduction of postsynaptic cortical activity, which would prevent recovery from amblyopia in monocular animals reared in standard conditions. In contrast, under EE conditions, the complex sensory stimulation exerted by this experimental protocol may cause an increment in the firing rate of the presynaptic thalamo-cortical neurons, leading to enhanced stimulation of postsynaptic cells in V1. Therefore, the modification threshold may be exceeded and, consequently, the strengthening of the synapses may favour recovery of normal visual functions. This may also explain the intriguing report that some adult amblyopic patients spontaneously improve vision in their amblyopic eye after loss of vision in the fellow eye. Humans, indeed, are certainly much more ‘enriched’ in terms of visual stimulation than rodents, which are nocturnal animals mostly relying to other senses, such as olfaction and touch.

The concept by which an increased sensory stimulation is necessary to trigger vision recovery in the long-term deprived eye is somewhat in contrast with previous findings showing an equal amount of increase in the percent of cells responsive to the deprived eye both in kittens which had the experienced eye enucleated at four months of age while the deprived eye remained closed and in kittens in which the previously deprived eye was opened for an additional three to four months [19]. However, it is possible that visual acuity recovery, differently from the pure number of neurons responsive to the deprived eye, which seems not to be affected by visual experience, is a process that requires the additional amounts of sensory stimulation provided by living in an enriched environment.

Finally, it is worth analysing our results with reference to previous data obtained by dr. M. Caleo and colleagues, which showed a strong contribution of callosal connections to visual cortex plasticity [28]. These authors demonstrated that, in rats monocularly deprived during the critical period, a continuous silencing of callosal inputs originating from the visual cortex contralateral to the open eye throughout the period of MD attenuates the OD shift, selectively enhancing deprived eye responses with no effect on the open eye-driven activity. A similar process may also have an effect in the experimental model analysed in the present paper. Indeed, our procedure of OND may lead to a robust reduction in the activation of the visual cortex contralateral to the open eye, leading to enhanced neuronal responses to the stimulation of the long-term deprived eye. This would facilitate recovery in V1, an effect consistent with the partial visual function improvements occasionally observed in amblyopic monocular humans. However, since it has been demonstrated that silencing of callosal inputs increases the response of visual cortical neurons only in a low-spatial frequency range, without any impact on visual acuity [28], the contribution given by reduction of callosal input strength prompted by OND on visual acuity recovery might be negligible.

### Conclusions

Amblyopic patients severely visually impaired in their non-amblyopic eye become nearly blind. Our results suggest that increasing levels of environmental stimulation may help trigger sight recovery in this unfortunate condition. The non invasive nature of EE renders this paradigm very promising in the field of amblyopia therapy in adult people. An open issue is to what extent is EE in animal models relevant for humans. EE is a complex paradigm, since an increased stimulation is provided at multiple sensory, motor, cognitive and social levels. Although most humans do experience a high degree of environmental complexity and novelty, levels of stimulation vary greatly among individuals and in different periods of life. Two types of enriched environment extensively studied in humans are perceptual learning and playing video games. Numerous papers have documented various and robust beneficial effects on visual functions elicited by these treatments in adult amblyopes after the end of the critical period [2], [29]. It remains to be investigated whether these paradigms based on active visual stimulation are also effective in triggering plasticity and vision recovery in monocular amblyopic people.
